# Dry Electrodes for Human Bioelectrical Signal Monitoring

**DOI:** 10.3390/s20133651

**Published:** 2020-06-29

**Authors:** Yulin Fu, Jingjing Zhao, Ying Dong, Xiaohao Wang

**Affiliations:** 1 Tsinghua Shenzhen International Graduate School, Tsinghua University, University Town of Shenzhen, Shenzhen 518055, China; fyl18@mails.tsinghua.edu.cn (Y.F.); wang.xiaohao@sz.tsinghua.edu.cn (X.W.); 2 Tsinghua-Berkeley Shenzhen Institute, Tsinghua University, University Town of Shenzhen, Shenzhen 518055, China; jjzhao2017@sz.tsinghua.edu.cn

**Keywords:** invasive microneedle electrode, surface electrode, capacitive electrode, electrode-skin interface impedance, bioelectrical signal acquisition

## Abstract

Bioelectrical or electrophysiological signals generated by living cells or tissues during daily physiological activities are closely related to the state of the body and organ functions, and therefore are widely used in clinical diagnosis, health monitoring, intelligent control and human-computer interaction. Ag/AgCl electrodes with wet conductive gels are widely used to pick up these bioelectrical signals using electrodes and record them in the form of electroencephalograms, electrocardiograms, electromyography, electrooculograms, etc. However, the inconvenience, instability and infection problems resulting from the use of gel with Ag/AgCl wet electrodes can’t meet the needs of long-term signal acquisition, especially in wearable applications. Hence, focus has shifted toward the study of dry electrodes that can work without gels or adhesives. In this paper, a retrospective overview of the development of dry electrodes used for monitoring bioelectrical signals is provided, including the sensing principles, material selection, device preparation, and measurement performance. In addition, the challenges regarding the limitations of materials, fabrication technologies and wearable performance of dry electrodes are discussed. Finally, the development obstacles and application advantages of different dry electrodes are analyzed to make a comparison and reveal research directions for future studies.

## 1. Introduction

**Figure 1:**
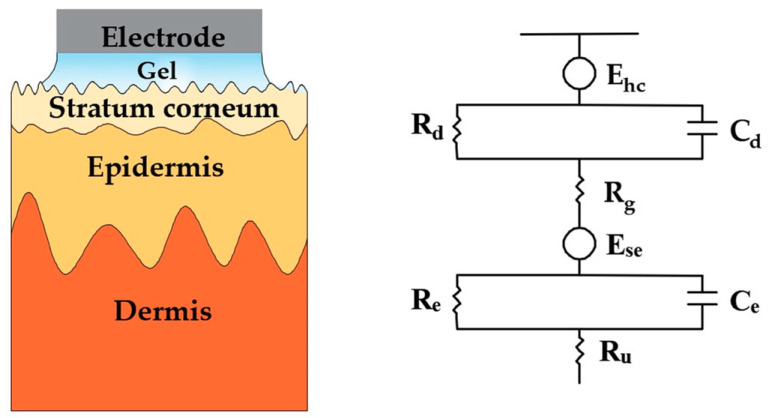
Schematic and electrical equivalent circuit model of electrode–skin interface for wet electrodes.

Living cells and tissues of human beings and animals, in both static state and active state, will generate regular electrical signals called bioelectrical signals. They can reflect the physiological state of organs or tissues and are important indicators to evaluate various physiological parameters of the body in clinical treatment, physical condition improvement, behavior analysis, etc [1]. For example, electroencephalogram (EEG) signals provide important diagnostic information for brain diseases such as epilepsy [2], dementia, and tumors; electrocardiogram (ECG) signals directly reflect the health state of the heart; and EMG signals are closely related to muscle fatigue and are often used to monitor the muscle status of athletes [3]. Consequently, the acquisition of high-quality signals is critical for these applications. It is worth stressing that all bioelectrical signals have several identical features: (1) small signal strength, whereby the amplitudes of these bioelectrical signals are generally below 5 mV (an EEG voltage amplitude is at the μV level); (2) instability and randomness [4,5], they are changing dynamically and external or internal stimuli will cause corresponding changes in human signals; (3) low signal-to-noise ratio (SNR) making them highly susceptible to interference [6]. In order to obtain stable, high-quality bioelectrical signals, the detection devices and operation process should meet the following requirements: (1) the electrode-skin interface impedance (EII) between electrode and skin should be minimized to ensure that the signal amplitude is large enough to be recorded clearly; (2) the electrode should provide a stable contact interface to attenuate motion artifacts during the detection; (3) the electrode should also have good biocompatibility without causing discomfort and chemical stability to avoid causing damage to the human body. The clinical standard Ag/AgCl wet electrodes, which is the most popular noninvasive electrode in clinical practice, are generally used with conductive gels as they reduce the electrode-skin interface impedance, which can provide an accurate and stable bioelectrical signal [7]. The sensing mechanism can be expressed by the simplified electrical equivalent circuit model shown in Figure 1. Simply, the electrodes represent an interface between the ionic charge transport in the physical body and the electron charge transport in the exterior detection device. A half-cell is formed at the electrochemical electrode–electrolyte interface when the wet electrodes are attached to the body. Here, the E_hc_ is the half-cell potential, and the structure of the interface is modeled by a capacitor C_d_ and a resistor R_d_ in parallel. The series resistance R_g_ is the effective resistance associated with interface effects of the gel between the electrode and the skin. A difference in ionic concentration across the stratum corneum results in a potential E_se_, which is given by the Nernst equation. The entire epidermis can be treated equivalently with a resistance R_e_ and a capacitance C_e_ in parallel. The dermis and subcutaneous tissues, mainly composed of blood vessels, nerves, preparatory glands, and hair follicles, their capacitance can be neglected and the impedance can be treated as a pure resistor R_u_. 

Benefitting from their excellent electrical properties, wet electrodes are widely used to collect bioelectrical signals, however, some shortcomings extremely limit the use of these electrodes, especially in long-time monitoring. Firstly, Ag/AgCl electrodes require skin preparation such as hair-cutting, dirt-removing, and electrolytic gel coating, which are quite time-consuming. Furthermore, the conductive gels may dehydrate and coagulate after long-time usage, which may increase the detection noise and reduce the signal quality. In addition, the gels have been observed to potentially cause allergic reactions or skin irritation in patients. For a long time, researchers have been looking for new solutions to overcome the shortcomings of wet electrodes without reducing signal quality. Dry electrodes without using conductive gels have the potential to address the above problems, as they have advantages in portability and convenience of use. In the signal acquisition with dry electrodes, they should both guarantee comfort properties in long-term detection and high quality signals for subsequent analysis, which is the final purpose of recent works. Compared to signals received from Ag/AgCl wet electrodes, signals received by dry electrodes have features of lower amplitude, higher impedance, more randomness and noise. Fortunately, the diverse materials and fabrication technologies available and the development of integrated electronics also open up more possibilities for dry electrode application. According to the principle of measurement, dry electrodes can be roughly classified into three types: invasive microneedle electrodes, surface electrodes, and capacitive electrodes [8]. 

Many previous works have reported on research on human bioelectrical signal acquisition systems. As early as 2000, Burke et al. reported a micropower dry electrode ECG preamplifier. The prototype amplifier is constructed on a matrix board with other circuits, and can be easily miniaturized. It can be self-sufficient because of its extremely low power consumption [9]. Meziane systematically reviewed dry electrodes used for ECG signal monitoring and compared the application of dry electrodes with different material substrates in biomedical and physiological studies [10]. Based on the different electrode structures, Lopez et al. reviewed the current research on dry electrodes for EEG measurement and provided some information about measurement methods and evaluation reports [11]. Specifically, Ren [12] and Acar [13] also reviewed and summarized the preparation technologies, mechanical properties and bioelectrical signal recording performances of the microneedle electrodes and fabric textile electrodes, respectively. However, despite the fact previous works that have made quite comprehensive summaries of the research on dry electrodes, they have not yet covered all types of dry electrodes, and there is no complete review of the research on dry electrodes used for human bioelectrical signal acquisition. 

This survey aims to present the main characteristics of the current dry electrodes developed for human bioelectrical signal monitoring, with a classification of materials, which currently represents the most critical aspect being dealt with. The survey is organized as follows: Section 2, Section 3 and Section 4 present invasive microneedle electrodes, surface dry electrodes and capacitive electrodes, respectively. In Section 5, the development of different types of dry electrodes is summarized, the applications of dry electrodes in some fields are illustrated, and the improvements that need to be made before dry electrodes are widely adopted are proposed.

## 2. Invasive Microneedle Electrodes

**Figure 2:**
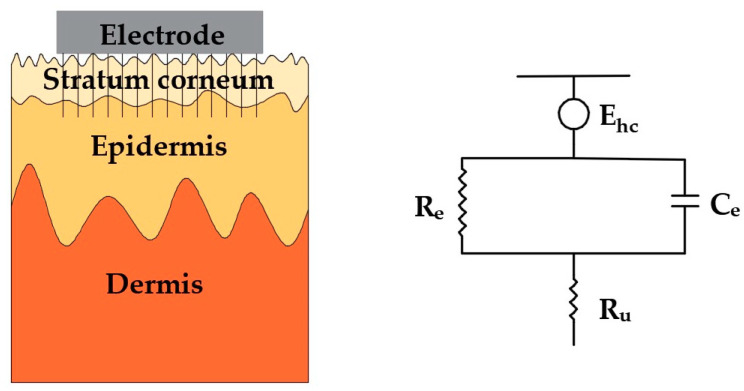
Schematic and electrical equivalent circuit model of electrode–skin interface for microneedle electrodes.

These microneedles can effectively minimize the influence of the unstable and insulating stratum corneum because the microneedles are in direct contact with the epidermis layer. Its simplified electrical equivalent circuit model is shown in Figure 2. It is clear that the equivalent circuit of invasive microneedle electrodes is relatively simple. In addition to the resistance of the conductive dermis and underlying tissues, only the coupling of the electrode and the conductive layers in epidermis is present, which is described by a half-cell potential E_hc_ and a resistor R_e_ and a capacitor C_e_ connected in parallel.

A microneedle array electrode is a promising dry electrode that has attracted increasing attention in ECG, EMG, and EEG monitoring due to its excellent properties including puncture without skin preparation, minimal skin trauma, low impedance, ease of operation, and high selectivity. In this section, we will introduce the previous microneedle electrodes focused on human bioelectrical signal monitoring [14,15], according to the preparation material(s) used. 

### 2.1. Silicon Material-Based Microneedle Electrodes

**Figure 3:**
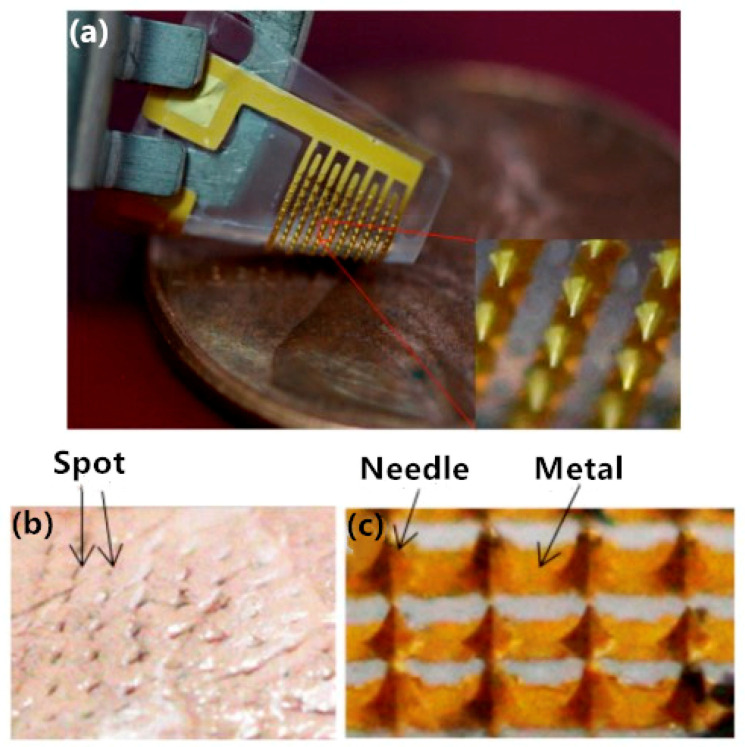
Photos and penetration test of microneedle array electrode: (**a**) flexible demonstration; (**b**) spots indicated that microneedle array electrode penetrated into tissue; (**c**) After penetration, microneedle array electrode retained previous structure and profile without obvious damage [19].

As a common material in microfabrication, silicon is often used as a substrate material for microneedle electrodes [16,17,18,19,20,21,22,23,24,25], and wet etch or dry etch photolithography technology is widely used in the fabrication of silicon-based electrodes. As early as 2001, Griss et al. [16] fabricated microneedle electrodes based on silicon that could be used for EEG signal acquisition. As shown in Figure 3, the electrode substrate was a 4 mm × 4 mm silicon wafer with a microneedle length ranging from 100 μm to 200 μm and diameters ranging from 30 μm to 50 μm. These microneedles could penetrate the stratum corneum of skin without reaching the dermis layer. 

Usually, rigid silicon materials have drawbacks in achieving conformal connection between electrodes and skin. To generate stable connections between the electrodes and the scalp, Wang et al. [19] proposed a flexible microneedle array electrode with a parylene substrate. Owing to the rigid silicon microneedles and flexible parylene substrate, impedance tests showed that the impedance density is about 7.5 kΩ/cm^2^ when the input signal frequency is 10 Hz, which is better than that of wet electrodes (10 kΩ/cm^2^).The excellent structure design and material choice make the electrodes retain low impedance density and conform to curved skin to ensure reliable EEG acquisition. The produced conical, mechanically robust microneedles on a flexible substrate could connect to the body conformably and significantly reduce any motion artifacts. Similarly, Zhang provided a method for establishing a microneedle array on a flexible PDMS substrate [21]. With a coated PEDOT/PSS conductive layer, the EII is lower than that of the general surface dry electrodes. More importantly, this method avoids costly techniques such as photolithography and dry etching technology (RIE, DRIE). By combining silicon needles and a flexible substrate, the electrode ensures the strength of the microneedles as well as good close adhesion with the skin. 

### 2.2. Metallic Material-Based Microneedle Electrodes

**Figure 4:**
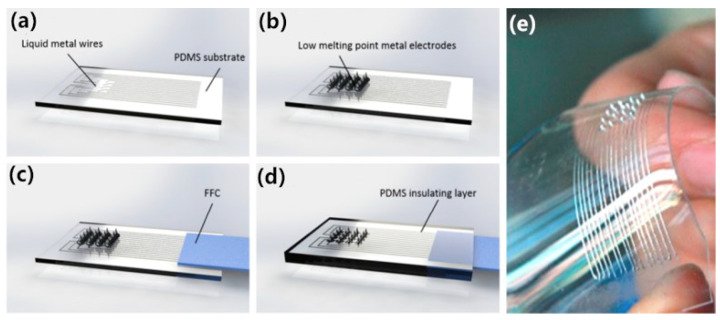
(**a**)–(**d**) Schematic illustration of the fabrication process of multi-channel flexible microelectrode array device. (**e**) Image of multi-channel flexible microelectrode [26].

To solve the shortcomings of silicon materials, researchers have considered other materials for microneedle electrodes. Metals can be adopted to fabricate microneedle arrays due to their good strength. Guo et al. [26] took some low melting alloys (Bi/In/Sn/Zn alloy, melting point only 57.5 °C) to make a flexible microneedle electrode based on PDMS (see Figure 4). The microneedle array was formed by dropping molten metal droplets directly onto the PDMS substrate. Here, the low melting point of the solid metal makes it easy to form microneedles and the rigidity of low melting alloys ensure effective skin piercing, while the flexible substrate guarantees the conformal contact with the skin at the same time. Impedance experiments show that the impedance value is comparable with the value of wet electrodes for a 10 Hz signal, but specific signal detection experiments have not to been carried out yet.

Currently, numerous methods for fabricating conductive patterns have been proposed, among which the magneto-rheological drawing lithography (MRDL) technique is a promising fabrication method that can easily draw a microneedle array from droplets of curable magneto-rheological fluid (CMRF) on flexible substrates under the assistance of an external magnetic field. In 2018 Ren et al. [27] proposed a microneedle electrode based on flexible PET film made by using laser-directed writing (LDW) technology and magneto-rheological drawing lithography (MRDL). The PET film substrate, which is extremely elastic, can be easily adhered to the skin surface. This electrode is able to collect more distinguishable features and mitigate the effect of motion artifacts during dynamical ECG recording.

### 2.3. Polymers Materials-Based Microneedle Electrodes

**Figure 5:**
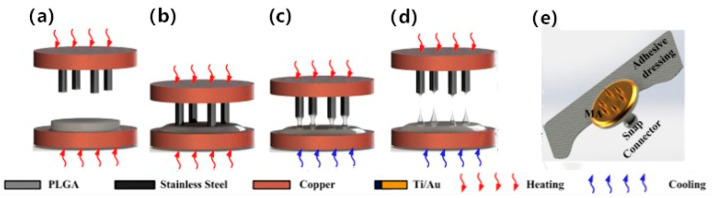
PLGA microneedle electrode (**a**)–(**d**): thermal drawing process of microneedle array, (**e**) the assembly of microneedle array electrode [28].

Apart from silicon and metal, many polymers such as poly (lactic-co-glycolic acid) (PLGA) [28], SU-8 photoresist [29,30] and PLA [31]) have also been proposed to fabricate microneedle arrays due to their good toughness and high biocompatibility. Magnetorheological stretch lithography, thermal drawing methods and 3D printing technology were employed to fabricate multiple microneedle electrodes. It’s worth noting that polymers need an extra conductive film (Ti, Au, Ag, or AgCl) applied by sputtering electroless plating, electrolysis, e-beam evaporation, etc. to enhance the electrical conductivity. For example, Ren et al. [28] succeeded in manufacturing a polymer-based microneedle dry electrode by utilizing the non-toxic nature of PLGA without producing environmentally harmful substances (see Figure 5). Thermal drawing can fabricate 3D polymeric microneedle array structures directly from viscous 2D polymeric materials, and the fabrication process is shown in Figure 5. The impedance test of the microneedle array electrode showed similar EII performance after piercing the stratum corneum compared with conventional wet electrodes, and the skin will return to its original appearance in a short time after the electrode removed. Thermal drawing technology is very simple, efficient, and low cost, it is also fit for various polymers and mass fabrication.

**Figure 6:**
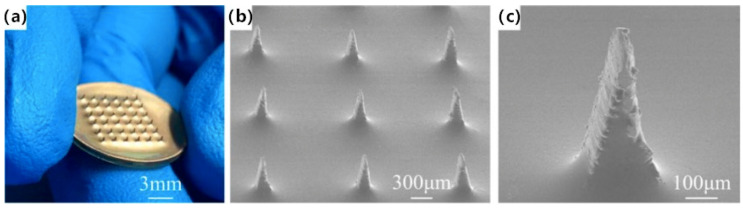
(**a**) Photograph of the CMAE; (**b**) SEM image of the CMAE; (**c**) SEM image of a single microneedle [30].

SU-8 photoresist is a negative photoresist used in lithography, which has good thermal stability, chemical stability and high mechanical strength after curing and it has been applied to microneedle fabrication. In this way, Sun [30] designed and fabricated a new composite microneedle array electrode (CMAE) to simultaneously record body temperature and ECG signals, as shown in Figure 6. The entire electrode consists of three layers, where the innermost layer is the microneedle structure layer (Ti), the middle one is the insulating layer (SU-8 photoresist), and the outermost layer is the conductive layer (100 nm-thick Au film). The SU-8 layer was used to insulate the Ti layer and Au layer. It produces a temperature-dependent voltage due to the thermoelectric effect, and the voltage is interpreted to measure the temperature. Results show that CMAE exhibits excellent mechanical properties such as good integrity after 100 cycles of insertion, indicating a reliable strength of the microneedles. The typical P-waves, QRS-complexes and T-waves of an ECG signal recorded by the CMAE were distinguishable. This novel CAME can be applied not only for ECG measurement, but also other physiological electrical signals and body temperature, indicating its broad application prospects in future human health monitoring.

In summary, silicon material-based microneedle electrodes show good results in terms of signal acquisition and fidelity in initial bioelectrical signal measurements. However, silicon-based microneedle electrodes also have several disadvantages, including sophisticated, costly, eco-friendly fabrication processes and fragility in use. These two disadvantages limit the application of silicon-based microneedle electrode in human bioelectrical signal acquisition. As for metallic materials-based microneedle electrodes, they show good microneedle strength during bioelectrical signal measurements. Metal is easy to form specific shapes and its high conductivity ensures the ability to record physiological signals accurately. However its poor biocompatibility makes it unsuitable for long-term monitoring. Polymers material-based electrodes on the other hand have good biocompatibility without causing any health issues, but these electrodes required an extra conductive film to enhance their conductivity, which means extra methods such as sputtering electroless plating, electrolysis must be adopted in the fabrication process. 

## 3. Surface Dry Electrodes

**Figure 7:**
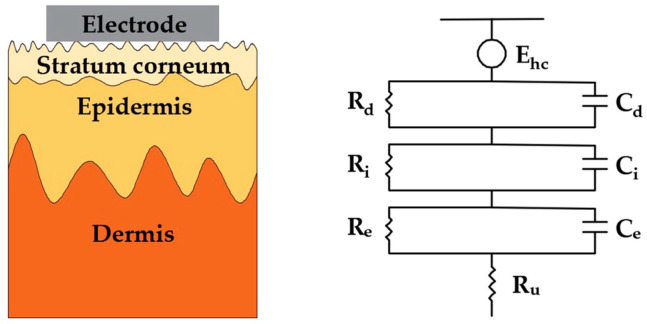
Schematic and electrical equivalent circuit model of electrode–skin interface for surface dry electrodes.

Surface dry electrodes are noninvasive electrodes with closely contact to the skin. They generally have a higher impedance than microneedle electrodes, but they won’t cause any discomfort to the human body. Their simplified electrical equivalent circuit model is shown in Figure 7. In contrast with conventional wet electrodes, there are no conductive gels between the electrode and the skin surface, so the resistance R_g_ is replaced by a capacitance C_i_ and a resistance R_i_, which are connected in parallel. Benefiting from their simple manufacturing processes and measuring principle, surface dry electrodes are the most studied dry electrodes so far. They can maintain a good contact with the skin, even during motion, and are thus suitable for use in ambulant situations, such as health-care and to monitor exercise. 

### 3.1. Metallic Material-Based Surface Dry Electrodes

Surface dry electrodes should be attached tightly to the skin when measure EEG signals, which means it is necessary to manually apply an additional force. Liao et al. [32] proposed a dry EEG electrode with several spring probes that allow high geometric conformity between the electrode and the irregular scalp surface. This probe-like electrode can adjust the additional force automatically using the spring located on the bottom of the electrode. The average correlation between the probe electrode and the wet electrodes measured at the forehead and the top of the head reached 95.26% and 91.47%, respectively, showing a good consistency in measuring EEG signals. 

To develop a practical dry electrode, the contact resistance between the dry electrode and the scalp must be addressed to construct an effective ion/electron conversion interface. In 2017, Song [33] put forward a multi-level EEG electrode with a three-layer electrode structure containing Ti plate, TiO_2_ nanotube, and a chitosan layer. The prepared Ch/Au-TiO_2_ dry electrode has an efficient and stable current conversion interface, a highly biocompatible contact surface and a fast electron transfer channel. Chitosan was chosen because it is a natural biopolymer with strong adsorption, anti-infectious properties and biocompatibility. The microscopic morphology of the electrode surface and the TiO_2_ nanotube structure improve the electron transfer performance and make up for the lack of interface electron capture caused by electrolyte deficiency, but on the other hand, it is clear that this composite electrode requires an extra bandage to fix it during measurement, which introduces the possibility of motion artifacts. Moreover, the flat electrode structure will make the EII increase significantly when measuring a hairy area. Based on these factors, the dry Ch/Au-TiO_2_ electrode developed in this study has great potential for use in a variety of applications, such as EEG, ECG acquisition, but significant improvements in the sensor are still needed before clinical application becomes a reality.

**Figure 8:**
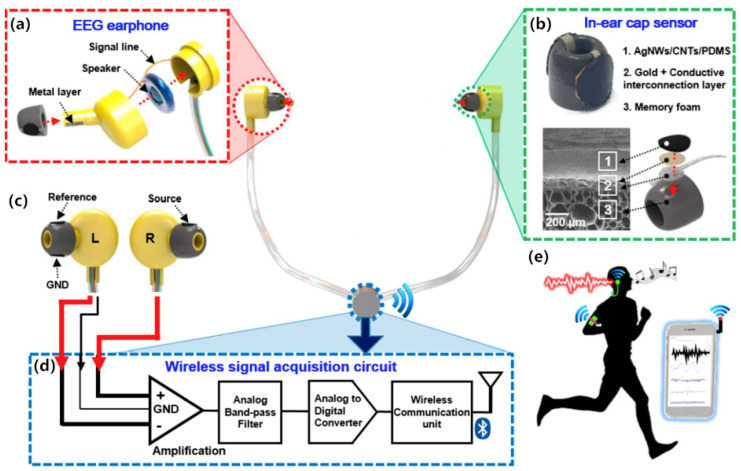
Components and design layout of a personal earphone for recording electrical brain activity. (**a**) Illustration of structural formation of portable earphones electrode; (**b**) Images of detailed structures and elements of fabricated earbud that consists of (1) AgNWs/CNTs/PDMS, (2) a conductive interconnection layer covered by a gold layer, and (3) supporting memory foam; (**c**) Illustration of the placement of each EEG electrodes (source, reference and ground electrodes); (**d**) Schematic description of signal processing block diagram; (**e**) The processed EEG data is displayed in real-time on personal devices screen such as a smart phone while in daily life activity [38].

It is obvious that the hair makes the contact interface between the surface electrodes and skin unstable, thereby affecting the measurement results. Researchers have tried to change the structure and material to reduce the influence. Fiedler [34] proposed a bullet-shaped electrode based on Ti/TiN in 2015. They minimized the impact of hair by reducing the contact area, Ti was chosen because of its good mechanical properties and biocompatibility, while a conductive TiN films layer showed high electrochemical stability and stable noise characteristics [35]. Besides, Kappel et al. [36] presented a novel dry-contact ear-EEG platform, comprising dry-contact electrodes and a soft-earpiece. Ear-EEG is a recording method in which EEG signals are acquired from electrodes placed on an earpiece inserted into the ear. Thereby, there is no need to worry about the effect of hair when measuring. The electrode is covered with a ruthenium oxide (IrO_2_) film to enhance the electrical conductivity, with epoxy adhesive applied on the backside of the construction. Although earphone-shaped electrodes [36,37,38] seem to be a perfect way for EEG acquisition without causing any pain and or risk of infection (see Figure 8), they still face the problem of motion artifacts caused by electrode-skin fitting.

Metal nanomaterials, such as AgNWs, are widely used to enhance the electrical conductivity in the fabrication of dry electrodes [39,40,41,42,43,44,45]. Myers et al. [39] developed a dry electrode based on AgNWs that can be used for ECG and EMG acquisition. In this work, an AgNWs/PDMS mixture worked as a stable conductive connection with a conductive metal snap. The results showed that the conductivity can reach 50 S/m under 50% tensile strain, due to the good conformal contact with the skin provided by the flexible AgNWs/PDMS electrode. Electrohydrodynamic (EHD) printing is an emerging printing technology that can be applied to the preparation of conductive electrodes. Cui et al. [42] prepared an AgNWs electrode by EHD printing. The AgNWs ink for EHD printing was deliberately developed with a conductivity of 5.6 × 10^6^ S/m. The EHD technique enables direct printing AgNWs on a diverse range of substrates, including PDMS, PET, glass, letter paper, nanofiber paper and polycarbonate filters. 

**Figure 9:**
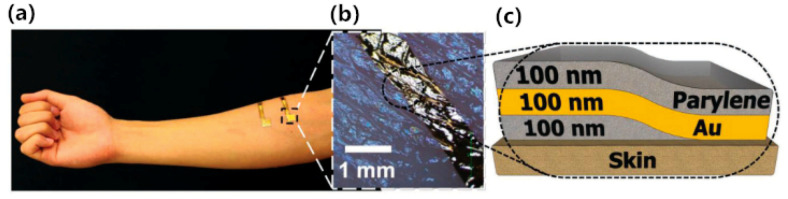
Skin-laminated sub-300 nm biopotential electrodes. (**a**) A photograph of 300 nm dry, thin film electrodes laminated on the skin of the subject. (**b**) A zoomed view of the sensor. (**c**) The structure of the thin film electrode laminated on human skin [46].

Thin, light-weight, and conformal bio-electrodes are highly desirable for bioelectrical signal monitoring. Also, self-adsorbancy is another desirable property that researchers have been pursuing. Nawrocki et al. [46] also proposed a self-adsorbing electrode of nanometer thickness, as illustrated in Figure 9. This novel electrode adopted a sandwich structure, where an Au film layer was sandwiched between two layers of biocompatible parylene. The thickness of each film layer is about 100 nm. Before placement on the skin, the skin-interface side of the sensor etched the parylene away, so it is possible to achieve directly contact between the Au film and the skin. This ultra-thin, low-noise, self-adsorbing dry electrode is simple in design and requires no extraordinarily complex technology, and the properties of the thin layers determine that they can achieve adhesion on complex three-dimensional surfaces, because their bending stiffness is similar to that of human skin, so it won’t cause discomfort. Compared with the standard Ag/AgCl wet electrode, this novel sensor exhibits motion artifact-less monitoring of EMG.

### 3.2. Carbon Material-Based Surface Dry Electrodes

Carbon-based materials (such as CNTs [47,48,49,50,51,52,53,54,55,56,57,58,59], graphene [60,61,62,63,64,65,66,67] or carbon black [68]) possess high mechanical strength and good electrical conductivity. To harness their unique properties, CNTs are often dispersed into a polymer matrix to fabricate electrodes. Gao [47] fabricated brush-like electrodes to improve comfort during testing. The bristle portion of the electrode is replaced by a carbon fiber material. After combination with PU, the PU-CNT mixture material increases the stiffness of the brush bristle. Finally, an Au film layer was electroplated on the carbon fiber bristles to enhance its electrical conductivity. The impedance was further reduced compared to the last brush-like electrode. PDMS has recently become a popular choice for biomedical applications for its non-toxicity, high gas and water permeability, and its amenability to a variety of fabrication methods. Jung et al. [50] proposed a dry self-adsorbing electrode made of CNT/aPDMS. The (CNT)/PDMS composite-based ECG electrode with a tiny vacuum chamber realized a self-adsorption function by the difference in air pressure between the inside and outside of the chamber. The new electrode has excellent electrical and mechanical properties, and it was noted that there was no skin irritation or itching after testing for one week. 

**Figure 10:**
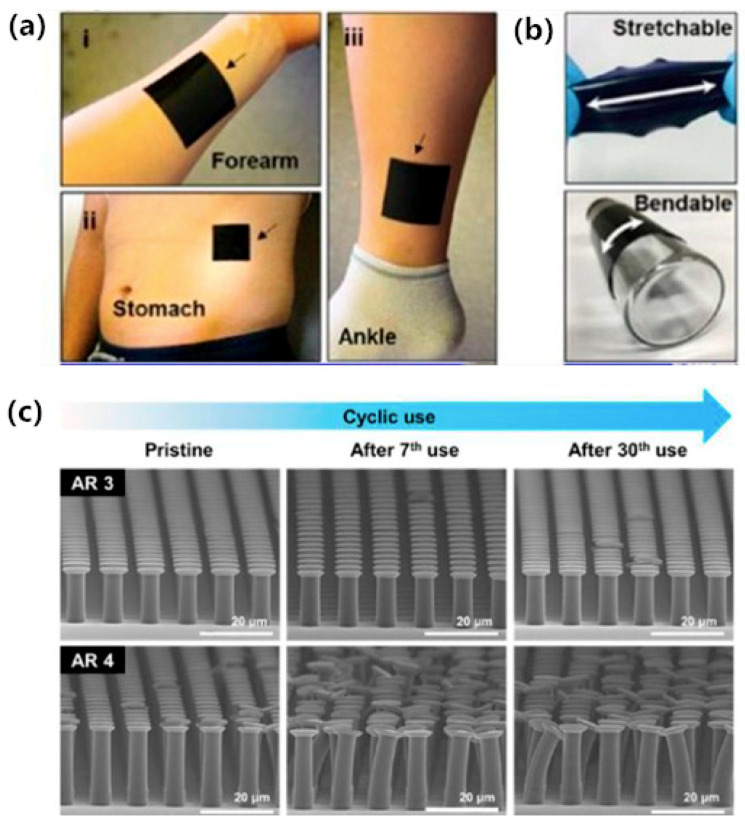
(**a**) Digital images of conductive dry adhesives conformally attached on human skin (i: foream, ii: stomach, iii: ankle); (**b**) Digital images of the stretched and banded conductive dry adhesives; (**c**) Degradation of structural integrity of microstructures with aspect ratio (AR) of 3 and 4 after cyclic use [53].

Self-adsorption has always been one of the goals pursued by researchers, as tape fixing and elastic strap fixing are not optimal. Despite the significance of developing a united, multifunctional component endowing both adhesively and conductivity, even in bending and stretching states, for practical applications, the current research and development trend for dry adhesives has focused only on a mimicking strategy for gecko-like structures and their adhesion properties. Kim et al. [53] proposed a novel flexible electrode based on the gecko-inspired hierarchical microstructure using CNT and graphene mixture (see Figure 10). The nano-mixture compensated for the bad conductivity of the small fraction of CNT. The ideal self-adsorbing electrode realized by the bionic gecko structure showed good cyclic adhesion (more than 30 times) with a normal adhesion of 1.3 N/cm^2^ on human skin. Moreover, the conductivity of the membrane electrode during the movement didn’t show a significant decrease, satisfying the requirements of long-time ECG measurement. Good self-cleaning ability introduced by its super-hydrophobic surface was another advantage, which may indicate a reusable all-in-one type electrode in ECG monitoring systems.

In addition, CNTs and AgNWs are outstanding conductive nanomaterials, so the combination of them is expected to have synergistic effects. Lee et al. [58] proposed a novel textile electrode fabricated by applying single-walled carbon nanotubes (SWCNTs) and silver nanowires (AgNWs) to a polyurethane (PU) nanoweb for monitoring ECG signals. However, the simple fabrication process of these electrodes is counterbalanced by a reduced robustness to noise and motion artifacts. Special attention is also required in long-term monitoring applications, as it may cause skin allergies, which could arise because of the presence of SWCNTs and AgNWs. Although the electrode has good ECG signal monitoring performance despite regardless of the motion, age and gender of the participants, some steps must be taken to enhance its biocompatibility.

**Figure 11:**
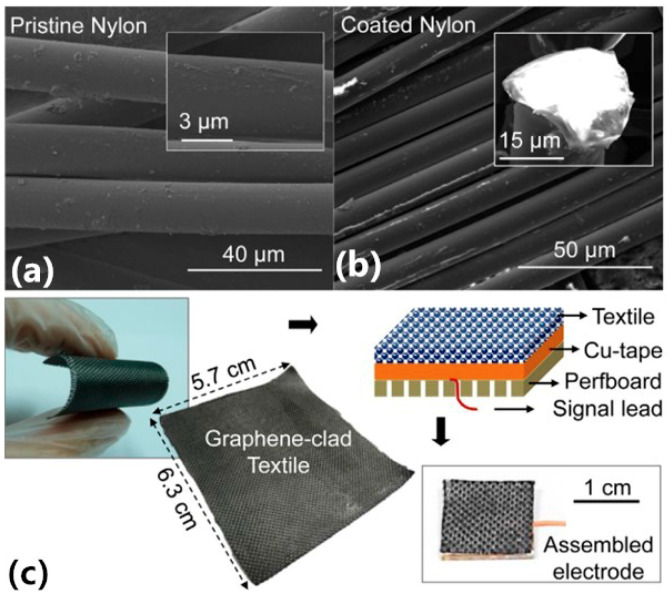
SEM showing (**a**) pristine nylon fiber; (**b**) graphene-coated nylon fiber; and (**c**) photograph of a sample of flexible nylon textile with rGO coating cut into smaller pieces and arranged in an electrode form for ECG testing [60].

Graphene-based electrodes also attract the interest of researchers because of their excellent electrical and thermal conductivity, and the high stiffness they display while being elastic. For example, Yapici et al. [60] developed graphene fiber electrodes with nylon fabric coated with graphene on the outer layer (see Figure 11). After a repeated immersion-drying-reduction process, the conductivity of the electrode changed from 6 × 10^-12^ S/cm to 4.5 S/cm. In the signal range of 10 Hz~1 kHz, the EII of the graphene-coated textile electrode is about 11.6 kΩ~87.5 kΩ, which is slightly larger than that of a general conventional Ag/AgCl wet electrode. Both the ECG signals and the power spectral density (PSD) of the ECG signals from the graphene clad textile and commercial electrodes were highly correlated after filtering. Overall, the graphene-clad textile electrodes were reliable, durable, and comfortable, and the fabrication process was suitable for mass production.

**Figure 12:**
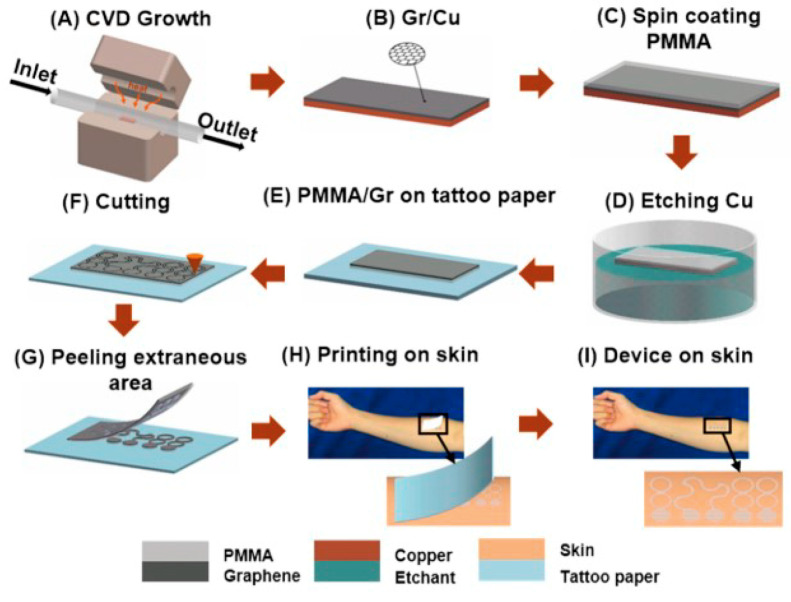
Fabrication process of a graphene electronic tattoo [62].

Tattoo-like epidermal sensors are an emerging class of wearable electronics, owing to their thinness and softness. Ameri et al. [62] developed a thin and soft graphene-based serpentine-like electronic tattoo electrode, the fabrication process is illustrated in Figure 12. The electrode can be directly attached to human skin during measurements with a high stretchability performance (over 40%), and the open-mesh structure provides good gas permeability. With the support of liquid bandage, it can realize long-term signal acquisition for several days. However, since the graphene layers obtained by chemical vapor deposition (CVD) technology are vulnerable, it is difficult to obtain the desired pattern and good durability. 

In 2018, Qiao [66] obtained a multilayer graphene electrode by a laser scribing method, which can grow and pattern graphene at the same time without a shield mask. Using laser to cut the transfer paper coated with graphene oxide, the high temperature generated by laser scribing can reduce graphene oxide to patterned graphene. The resulting electrode can be adsorbed on hairy or moving skin and maintains a stable impedance after 1000 cycles, making it extremely durable. Both of these graphene electrodes are very thin and soft, and can be well-fitted to the skin, significantly reducing the effects of noise such as motion artifacts. 

### 3.3. Polymer Material-Based Surface Dry Electrodes

In order to avoid the problems caused by irregular skin, surface EEG electrodes are usually designed into many shapes, such as bullet-shaped electrodes, probe-shaped electrodes [69,70,71], earphone-shaped electrodes, etc. However, stiff substrate dry electrodes will be uncomfortable or even hurt the skin when applied in daily life. Moreover, the stiff substrate will also lead to bioelectrical signal distortion due to motion effects. Lin et al. [72] began to develop a loose-like foam electrode. The dry foamed electrode consisted of a conductive polymer foam whose surface was covered by a partially polarizable conductive fabric. The foamed electrode provided a high degree of geometric consistency between the electrode and the irregular scalp surface and could maintain a low EII, even under motion. In addition to measuring EEG directly in hairy areas, we can also measure EEG in hairless areas. 

Norton et al. [73] introduced a collapsible flexible electrode that can be attached to the auricle of human body to achieve long-term, high-quality EEG measurements. The electrode was made of polymethyl methacrylate, Au and polyimide. Like ear-phone sensors, it just placed on the ear during measurement, and the serpentine pattern can reduce the effective elastic modulus of the electrode to provide excellent deformability performance. This flexible conformal system can be applied to the skin for more than two weeks, which was superior to other methods for EEG acquisition. In recent years, flexible and highly integrated epidermal electronic systems that can be directly attached to the skin through van der Waals forces have attracted much attention. 

**Figure 13:**
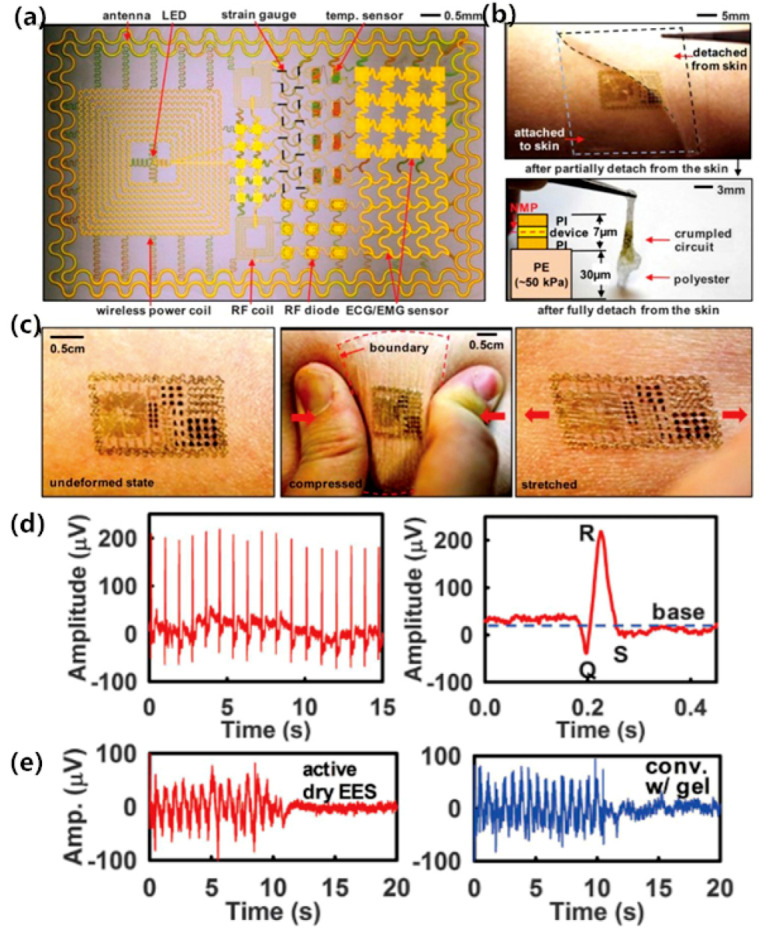
(**a**) Image of a demonstration platform for multifunctional electronics with physical properties matched to the epidermis; (**b**) Multifunctional electronics peel away from the skin; (**c**) Multifunctional electronics on skin: undeformed (left), compressed (middle), and stretched (right); (**d**) ECG signals measured with an active EES attached to the chest (left), and magnified view of data corresponding to a single heartbeat (right); (**e**) (Left) EMG measurements using an active EES, mounted on the right leg during simulated walking (from 0 to 10 s) and standing (from 10 to 20 s). (Right) Recordings collected with conventional sensors and conductive gel. [74].

**Figure 14:**
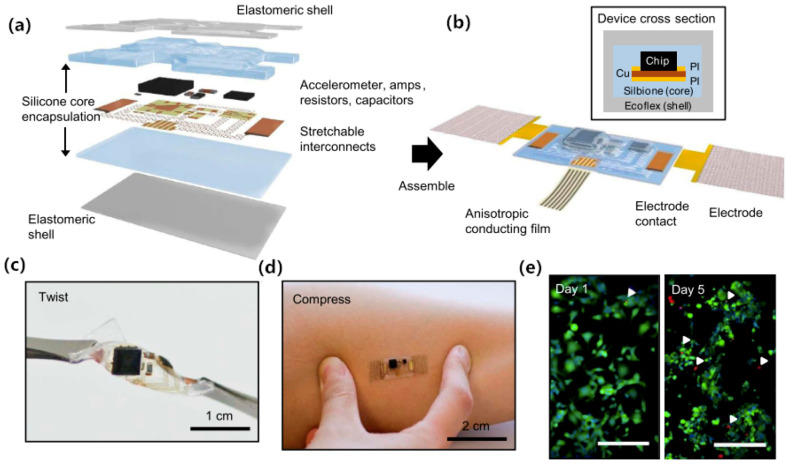
(**a**) Exploded view diagram of the overall design structure of the system; (**b**) Illustration of the assembled device; (**c**) Device held by tweezers in a twisted configuration; (**d**) Device mounted on skin while compressed by pinching; (**e**) Fluorescence micrographs of cells cultured on the surface of a device to illustrate its biocompatibility [75].

Many of the epidermal electronic systems are thin, lightweight, low-modulus, and skin-compatible architectures that can ensure high-fidelity mechanical coupling across the skin/device interface and multifunctional measurement, they showed great potential for human-machine interfaces. Kim [74] proposed a tattoo electrode that integrates electrophysiological sensors, temperature sensors, and strain sensors, transistors, light-emitting diodes, photodetectors, radio frequency inductors, capacitors, oscillators, and rectifying diodes (see Figure 13). Such a highly integrated electrode system reduces the size of the whole measurement system, meanwhile it can be connected to smartphones for real-time monitoring, which would greatly improve the convenience of ECG measurements. Similarly, Liu [75] demonstrated a mechanical acoustic sensor that can directly attached to the skin. It can simultaneously record ECG and SCG (seismocardiogram) data using a miniature accelerometer (see Figure 14). This core/shell structure decreases the physical constraints for motions, and the low-modulus structure maintains a high degree of geometric consistency with the skin, showing advantages in signal acquisition during moving activities. By modifying the circuit and chipset, the mechanical acoustic sensor was also expected to provide wireless power and communication functions, demonstrating an efficient, continuous and non-invasive ECG monitoring method.

PEDOT: PSS, a well-known conducting polymer, can be easily processed into films using traditional techniques such as inkjet printing, brush printing, and screen printing. Recently, PEDOT:PSS has been successfully integrated into textiles (cotton, polyester, nylon, etc.) [76,77,78,79,80] or paper-based materials [81]. PEDOT:PSS coatings were used on the surface to enhance the electrical conductivity and reduce EII. Sinha et al. [77] used screen printing technology to fabricate a textile electrode that was directly integrated into a sports tights to achieve ECG acquisition. After five times printing, the electrode surface resistance can be as low as 5.6 Ω/sq. The presence of the tights ensures that ECG signals can be acquired at a heart rate of 180 bpm and the acquired signals are highly consistent with wet electrode results. A smart clothing integrating the electrode, wire and other modules could improve the sustainability and convenience of health monitoring.

**Figure 15:**
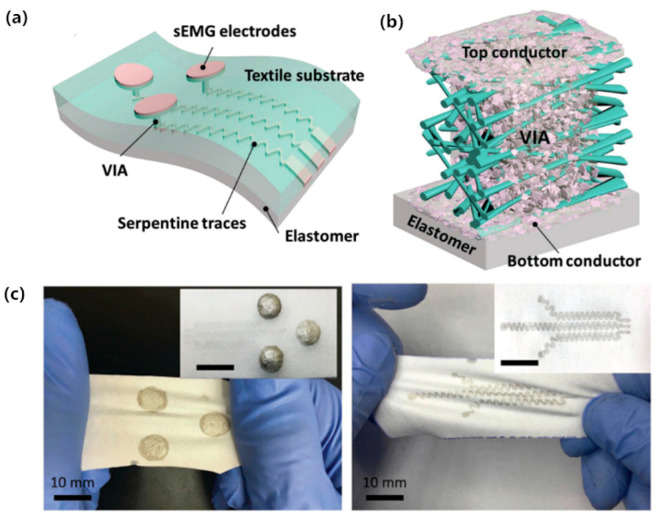
Two-layered printed e-textile patches. (**a**) Schematic of the e-textile patch comprising three printed electrodes, VIAs, and serpentine traces encapsulated by dielectric elastomer (PDMS, or acrylics); (**b**) An illustration of the VIA; (**c**) the sensory electrode and the serpentine traces sides [82].

The electrodes mentioned above are independent measuring electrodes, however, electronic products that integrating conducting polymer into clothing have emerged as a promising candidate for wearable personal healthcare applications [82,83,84,85,86,87,88]. La et al. [82] formed a single layer of conductive fabric by controlling penetration of Ag-particle/fluoropolymer composite ink into a porous textile (see Figure 15). The base material of the electrode is nano-sized PU fiber textile, and the well coated by composite conductive ink improves the electrical conductivity of the fabric (3200 S/cm). Further, after stretching the electrode for 1000 cycles under a state of 30% strain, the electric resistance was only increased by 5-fold, showing excellent tensile properties. Apart from this, the sensing electrode and the conductive circuits for signal transmission are respectively printed on both sides of the fabric substrate, indicating a vertical conductive path. Finally, a surface EMG system with wireless data transmission was obtained by integrating the electrode with the amplifier module, filter module, ADC conversion module and Bluetooth transmission module. The spectrum of the bandpass filtered EMG signal shows that most of the signal components fall between 60~150 Hz, which is consistent with the characteristics of EMG signals.

Metallic materials are not common materials used for surface dry electrodes fabrication due to the high cost, combination problems with flexible substrate and time-consuming preparation process. In contrast, CNTs and graphene are the most used materials, because of their high mechanical strength, good electrical conductivity, and mass producing ability at a relatively low cost. Although both of them are currently the most widely used electrode materials, there are still many improvements to be made, such as reducing the harm of CNTs to the human body and making graphene electrodes easier to fabricate through technological improvements.

## 4. Capacitive Electrodes

**Figure 16:**
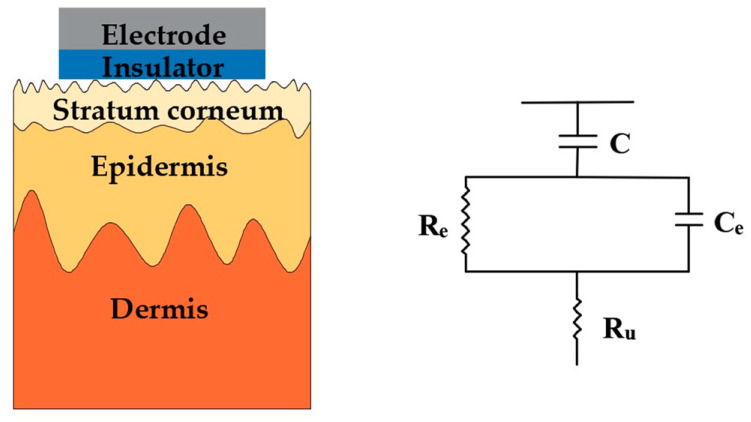
Schematic and electrical equivalent circuit model of electrode–skin interfaces for capacitive electrodes.

Since Lopez and Richardson first proposed the concept of capacitive electrodes in 1969 [89], these electrodes have been applied on various physiological signals acquisition in the past 50 years. The working principle of the capacitive electrode is quite different from the above two types of contact electrodes. It is equivalent to a capacitor coupled to the skin surface, it doesn’t have to be in close contact with the skin surface, which can greatly improve the comfort. Its simplified electrical equivalent circuit model of surface dry electrodes is shown in Figure 16. It coupled with the skin in a noncontact manner with air, clothing, or other materials between the electrode and the skin. Therefore, impedance contribution of the air/clothing or other dielectrics can be described by a capacitance C, and the rest part is same as that of conventional wet electrodes.

The common capacitive electrodes usually integrated with the front-end amplifier circuit on PCB [90,91] or copper plate [92] to realize bioelectrical signal acquisition. Except the conventional electrodes, many designs that getting closer to our daily life were developed. For example, Lim [93] and Steffen [94] have designed ECG electrodes in combination with office chairs, so they sit on the office chair to complete the ECG test. Varadan [95] and Rai [96] embedded their electrodes into fabrics, sports vests and T-shirts, which can achieve ECG detection during exercise, with the help of smart phones, real-time monitoring can be realized in their works.

Chen et al. [97] proposed a relatively simple non-contact electrode. The electrode plate is a copper piece with a diameter of 20 mm and a thickness of 2 mm, supplemented by an active circuit module. The active circuit module includes a front-end amplifier and a back-end filter. Not only that, but the research team also created a mechanically adaptive device that uses springs to hold the electrodes in place, which mitigates artifacts caused by motion. The correlation between the EEG signals in the hairy site obtained by the wet electrodes and the capacitive electrodes reaches 92.05%, and the mechanical adaptive device can reduce the generation of motion artifacts to improve the quality of the signal. Moreover, in an experiment of 5 hour duration, the experimental result of the wet electrode deteriorates as the conductive gel dries, but the changes of the capacitive electrode are negligible.

Many EEG capacitive electrodes have rigid surfaces that produce an undefined contact area due to their stiffness, which renders them unable to conform to the head curvature and locally isolates hairs between the electrode surface and scalp skin, making EEG measurement through hair difficult. In 2012, Baek et al. [98] proposed a polymer foam electrode with a metal film on its surface for EEG signals acquisition. This enabled EEG measurement through hair without any conductive contact with bare scalp skin. These foam-type electrodes are well-suited for contacting between the scalp and the electrodes and can be placed on a hat or helmet. Although the signal quality is not as good as that obtained with conventional wet electrodes, it is already superior to that of ordinary dry electrodes. 

Four years later, Lee proposed a CNT/aPDMS electrode that is elastic, highly conductive, self-adhesive, and capable of making conformal contact with and attaching to a hairy scalp [99]. The structure was a three-layer structure: a disc layer, a PDMS ring, and a combination of CNT and an adhesive polydimethylsiloxane (aPDMS) layer. The electrode made conformal contact with the hairy scalp, and the air gap between the electrode and the scalp was replaced by a conductive CNT/aPDMS layer that transmitted the EEG signals to the electrode around the hairs. Although the production process is cumbersome, the resulting signal quality, including sensitivity to motion artifacts, is comparable to that of conventional wet EEG electrodes. Also, the electrode can be reused without the need for an additional preamplifier or any gel, so it can be widely used in EEG measurements.

Stable acquisition of electrocardiograph (ECG) /electromyography (EMG) signals is critical and challenging in dynamic human machine interaction. Lee et al. [100] proposed a capacitive electrode packaged by PDMS that can be implanted under the epidermis. A four-week test of implanting the electrode into the subcutaneous tissue of the mouse revealed that the electrode recorded a high-quality ECG signal. However, the high cost in electrode fabrication and the requirement of wires for connecting to the subcutaneous electrodes during measurement makes them susceptible to infection. Huang et al. [101] designed a self-adsorbing capacitive electrode using PDMS as a substrate material. The electrode possessed a sandwich structure, with a 0.3-micron Au film sandwiched between two 1.2-micron PI films. By changing the line width of the intermediate Au film layer, the scale factor and the aspect ratio the tensile properties and measurement properties of the electrode changed. The high geometric consistency between the electrode and the skin greatly reduces the generation of motion artifacts and improves the sensitivity and stability of the signal.

## 5. Summary and Prospects

In this paper, dry electrodes for the acquisition of bioelectrical signals, including ECG, EEG and EMG, have been reviewed. These dry electrodes can be divided into three types according to their working principles: invasive microneedle electrodes, surface electrodes and capacitive electrodes. The research status of each type is summarized in Appendix A Table A1, Table A2 and Table A3, respectively.

The invasive microneedle electrodes have the lowest contact impedance among the dry electrodes, even lower than that of the conventional wet electrodes. The reliable contact interface between the electrode and the skin, result in the similar signal quality to the wet electrodes. However, penetrating the stratum corneum of skin increases the possibility of infection, and the length of microneedles should be appropriate to penetrate the stratum corneum without breaking it or causing pain, which limits the applications of microneedle electrodes. Besides, most microneedle electrodes are based on stiff substrates such as silicon and metallic materials, making the electrode more susceptible to motion artifacts. Therefore, the microneedle electrode is unlikely to become a future development direction in dry electrodes.

While the surface dry electrodes can achieve a close fit between the electrode and the skin without penetrating the skin, guaranteeing a noninvasive detection method, however, motion artifacts between the electrode and the skin remain a major problem during long-term signal collection. Adhesives that could maintain adequate adhesion on skin and nanostructure patches that possess mechanical interlocking, nanopillars or 3D architectures inspired by nature are proposed to solve this problem and show promising developments. Such adhesives, however, cannot maintain adequate adhesion on wet skin or skin under flowing water. Nanopillar patches have shown striking adhesive performance through mechanical interlocking. For example, previous studies specified that concave nanopillars covering the hexagonal micropatterns increase attachment to wet surfaces self-adsorption, this idea was inspired by tree-frogs [102]. Meanwhile, the 3D architectures of octopus suckers have been studied to fabricate surface electrodes [103]. The convex shaped architecture in its suction chamber, in particular, has demonstrated remarkable adhesive performances not only on dry surfaces but also on wet and rough surfaces [104].

Capacitive sensors, which need no physical, conductive contact with the skin, will be more reliable and comfortable than other type sensors in some case. For example, capacitive sensors are unaffected by any perspiration film between the electrodes and the skin. Besides, the signal distortion can be significantly decreased by the high input impedance introduced by the signal amplification. But comparing with wet electrodes, capacitive measurement is confronted with the ultra-high impedance and the instability of capacitive electrode-skin interface, which lead to the susceptibility to noise and motion artifacts. In order to achieve high quality signal acquisition, the following points are main strategies to acquire high quality signals: (1) electrode impedance matching; (2) stable electrode-skin interface (i.e. installation of the electrodes); (3) ultra-high input impedance; (4) high Common Mode Rejection Ratio (CMRR) and high SNR. Although the capacitive measurement of human bioelectrical signals has made promising progress in the recent decade, there are still room for improvement to replace the traditional wet electrodes in current stage. 

To conclude, among these three types of dry electrodes, it is clearly that the EII of the microneedle electrodes is the smallest, while that of the capacitive electrodes is the largest, according to their measurement principles. However, the limitations of microneedle electrodes have impeded the development of microneedle electrodes. The capacitive electrode has a unique advantage when measuring EEG, as it does not need to contact the surface of the scalp and the capacitive electrode can also be integrated into clothing or other fabrics to make a wearable smart textile, which is convenient and quick to achieve signal monitoring. In contrast, the flexible surface electrode has the characteristics of good contact, convenient measurement, simple structure, etc., which will greatly improve the contact interface between the electrode and the skin, significantly reduce the motion artifacts and noise of the signal, and obtain the human physiological electrical signal with a high SNR. Predictably, these well-developed surface electrodes would present an alternative to the traditional Ag/AgCl wet electrodes in future clinical practice.

The dry electrodes for human bioelectrical signal acquisition introduced in this paper are superior to conventional Ag/AgCl wet electrodes in terms of time consumption, using comfort, and even measurement performance, they have been applied in some areas. First of all, the dry electrode measurement for human bioelectrical signals is promising, although there still needs to be more efforts on reducing the gap between current technique and clinical diagnostic standard. Some studies have tested their system with clinical devices, proving the feasibility for future clinical application [105,106]. Secondly, dry electrodes are used for daily health monitoring and sports monitoring. The long-term monitoring of human bioelectrical signals is generally required for both patient health and rehabilitation. Studies have been proposed for human bioelectrical signals monitoring with the electrodes placed on the seat [107], sports bra [108], or textile [75,76,77,78,95], which make health monitoring easier. Besides, dry electrodes also used for sports monitoring, Pino et al. [109] proposed a shirt for athlete training, the hardware acquires EMG signals from six channels and transmits them wirelessly to a PC via Bluetooth. In the PC, EMG signals are analyzed to give feedback to the user regarding the exercise being performed. The system is able to monitor EMG activity and provide valuable information in real-time for professional and amateur athletes and their sports coach, improving their training protocols. Human-machine interface (HMI) is a user interface that connects an operator to the controller for an industrial system. The human bioelectrical signals, such as EEG, EMG, ECG, or other electrical potentials in living bodies, may serve as essential inputs for active control of prosthetics or for HMI systems [110]. Among them, the brain-computer interface, is an important example of how to acquire EEG signals as the input for various applications, augmenting and repairing human cognitive or sensory-motor functions. 

In a word, dry electrodes have comparable performances to wet electrodes and they are experiencing booming development. However, wet electrodes still occupy the main market, and the dry electrodes have not realized real marketization due to the many problems that still exist. Signal processing, circuit design, manufacturing process and the size of the integrated device are desired to have a mature solution in dry electrodes’ development, but we believe that dry electrodes with superior performance and more convenient operation will play more and more important roles in the daily monitoring of bioelectrical signals.

## Appendix A

**Table A1:** Summary of the invasive electrode situation.

References	Description	Materials	Type	Abstract	Technology
Griss [16]	Microneedles array electrodes coated with silver/silver chloride	Silicon, silver/silver chloride	Invasive EEG electrode	The lengths and diameters of microneedles range from 100 to 210 μm and 30 to 50 μm, respectively. The impedance remains about 18 kΩ at 10 Hz.	DRIE, wet etching, evaporation, thermal oxidation
Dias [18]	54.7°-angle microneedles array and wireless system	Silicon, iridium oxide	Invasive EEG electrode	The electrode is composed by 16 microneedles that are fabricated by wet etching.	Wet etching, sputtering
Wang [19]	Rigid microneedles array on flexible substrate	Silicon, Parylene, Cr/Au	Invasive EEG electrode	The experimental results show that EII is lower than that of the conventional wet electrodes in EEG frequency domain.	LPCVD, wet etching, lift-off process, sputtering
O’Mahony [20]	Silicon -based Microneedle electrodes	Silicon, Ag	Invasive ECG electrode	The electrode consisted of a 5 × 5 arrangement of 300μm tall needles located at a pitch of 1.2 mm on a 7 mm × 7 mm die.	Anisotropic etching, thermally evaporation
Hsu [21]	Barbed microtip-based electrode arrays	Silicon, Ti/Ag	Invasive ECG electrode	KOH anisotropic wet etching was employed to form a standard pyramidal microtip array and isotropic etching was used to fabricate barbs on these microtips.	Anisotropic etching, isotropic etching
Matteucci [22]	Microneedle dry electrode built with deep X-ray lithography	Silicon, Au/Pd, Cu	Invasive EEG electrode	The electrode is high aspect ratio microelectrode with hollow microneedle arrays.	Deep X-ray lithography, Soft lithography, LIGA, pulsed laser deposition, evaporation
Zhang [23]	Silicon microneedles array with sharp tips	Silicon, PEDOT/PSS, PDMS	Invasive ECG electrode	Silicon microneedles are fixed on the PDMS substrate through bonding. PEDOT/PSS further decrease the EII.	Dicing saw, isotopic etching, dip coating
Forvi [24]	Microneedles-based dry electrodes	Silicon	Invasive EMG electrode	This dry electrode is fabricated by anisotropic wet etching technique. It is 10 mm square arrays hosting 8×8 pyramidal microneedles, the impedance value obtained after piercing is near to 5~10 kΩ.	Anisotropic etching
Lin [25]	Self-stabilized diamond-shaped microneedles array	Silicon, Au	Invasive EEG electrode	The length of microneedles is about 250 μm, the impedance of electrode is about 5 kΩ at 10 Hz.	Anisotropic etching
Guo [26]	Low melting point metal-based flexible 3D microneedles array	PDMS, metal	Invasive EEG electrode	The electrode has a flexible PDMS substrate, was based on low melting point metals, and it can be stretched to a maximum of 42% before it becomes non-conducting.	Phase transition method, 3D printing
Ren [27]	Flexible microneedle array electrode for bio-signal monitoring	Epoxy novolac resin, iron particles, Ti/Au, polyimide	Invasive ECG electrode	Microneedle array can be one-step drawn from the droplet array of curable magnetorheological fluid under the assist of external magnetic field. Ti/Au film was coated on the surface to insure the conductivity.	Magnetorheological drawing lithography, magnetron sputtering
Ren [28]	Microneedles array fabricated by thermal drawing	PLGA, Ti/Au	Invasive EEG electrode	The electrode is composed of 6 × 6 microneedles with an average height of about 500 µm. It presents less variation of impedance and better stability.	Thermal drawing method, magnetron sputtering
Srivastava [29]	SU-8 microneedles based dry electrodes for EEG	SU-8 negative photoresist, Au	Invasive ECG electrode	The electrode is fabricated by the UV maskless lithography in specially-made molds using a biocompatible polymer SU-8 photoresist.	Magnetron sputtering, UV maskless photolithography
Sun [30]	Composite Microneedle Array Electrode	Ti, SU-8 negative photoresist, Au	Invasive ECG electrode	The electrode consists of a 6 × 6 microneedles array with a height of 500 µm and a base diameter of 200 µm.	Spinning coating, sputter coating, laser cutting

**Table A2:** Status of invasive electrodes.

References	Description	Materials	Type	Abstract	Technology
Liao [32]	Surface electrode with 17 spring contact probes	Stainless-steel, Au, Cu, BeCu	Surface EEG electrode	The lengths and diameters of microneedles range from 100 to 210 μm and 30 to 50 μm, respectively. The impedance remains about 18 kΩ at 10 Hz.	DRIE, wet etching, evaporation, thermal oxidation
Song [33]	Chitosan/Au-TiO2 nanotube-based dry electrodes for EEG	Chitosan (Ch), Au, TiO_2_ nanotube, Ti	Surface EEG electrode	This dry electrode is a Ch/Au-TiO_2_/Au-Ti multilayer film, the mean impedance values were approximately 169 ± 33.0kΩ at 2.15Hz and 67.4 ± 8.9 kΩ at 100 Hz.	Electrochemistry-based multi-potential step technology, electrochemical anodic oxidation method
Fiedler [34]	Novel multipin electrode cap system	Polyurethane, Ag/AgCl	Surface EEG electrode	The electrode consists of 24 single pins with circular tops of 1 mm in diameter and a height of 6 mm, the distances of the pins are 2.5 mm.	Not mentioned
Kappel [36]	A novel dry-contact ear-EEG electrode	Ti, IrO_2_, silver epoxy, acrylic plastic	Surface EEG electrode	This earphone electrode doesn’t need to measure on the hairy sites, but the change of the within-ear configuration resulted in low SNR.	Thermal oxidation, casting
Lee [38]	Personal earphone electrode for EEG	AgNW, CNT, PDMS.	Surface EEG electrode	The structures and elements of fabricated earphone that consists of AgNWs/CNTs/PDMS layer, a conductive interconnection layer covered by a gold layer, and supporting memory foam.	Not mentioned
Myers [39]	AgNWs dry electrode	AgNW, PDMS	Surface ECG electrode	AgNWs with average diameter of 90nm and length of 10~60 mm, and the conductivity of the electrode is over 50 S/m.	Casting
Cui [42]	Electrohydrodynamic printing AgNWs electrode	AgNW, PET, PDMS, paper	Surface ECG electrode	After post treatment, printed AgNWs showed an electrical conductivity as high as ∼5.6 × 10^6^ S/m.	Electrohydrodynamic printing
Nawrocki [46]	Self-adhesive and ultra- conformable, sub-300 nm dry thin-film electrode	Parylene, Au	Surface EMG electrode	This dry electrode is sub-300 nm thin film electrode that is self-adhesive and conformable to complex skin surfaces.	Spin coating technique, thermally deposition
Gao [47]	Soft pin-shaped dry electrode with bristles	PDMS, CNT, PU, carbon fiber, Au	Surface EEG electrode	The diameter of the pedestal was 17 mm, and its thickness was 7 mm, the impedance was 10–100 kΩ order of magnitude.	Magnetic stirring, electroplating, casting
Lee [49]	CNT/PDMS conducting thin film electrode	CNT, PDMS	Surface ECG electrode	With 1.5 wt% the CNT dispersion, a flexible film was successfully tested for long-term usage as an ECG electrode.	Two-step dispersion method, spinning
Jung [50]	CNT/PDMS Composite Flexible Dry Electrodes	PDMS, CNT	Surface ECG electrode	The signal quality depended on the composition of the CNT/PDMS composite, and on the size of the electrode.	Two-step dispersion method, Casting
Peng [51]	Flexible micropillars electrode based on carbon nanotube/polymer hybrid	PDMS, CNT	Surface ECG electrode	The diameter and height of the single micropillar are 50 μm and 100 μm, respectively. Its EII is lower than that of the flat electrodes.	Spinning, UV photolithography, casting
Kim [53]	Dry electrode based on 1D−2D hybrid carbon nanocomposites	Graphene, CNT, PDMS	Surface ECG electrode	The electrode shows the lowest volume resistance (∼100 Ω·cm) at an optimized filler ratio with a normal adhesion force of ∼1.3 N/cm^2^ on human skin, which is comparable to that of commercial wet adhesives.	Casting
Yapici [61]	Graphene-clad textile electrodes	Graphene, textiles	Surface ECG electrode	The textiles electrode based on scalable and robust synthesis of conductive fabrics with graphene cladding. The EII ranges from 87.5 kΩ to 11.6 kΩ.	Dipping and coating, thermal treatment
Lou [62]	Flexible Graphene Electrodes	Graphene, PET, Ag, polyester fiber	Surface ECG electrode	The graphene textile electrode demonstrates comfortability, good biocompatibility, and high electrophysiological detection sensitivity.	Chemical vapor deposition, chemically reduction
Das [64]	Chemically reduced graphene oxide-based dry electrodes	Chemically reduced graphene oxide	Surface ECG electrode	The surface resistivity of the electrode is found 28 Ω/sq.	Chemically reduction, heating
Karim [65]	Inkjet-printed graphene-based textile	Graphene ink, textile	Surface ECG electrode	Inkjet printing reduces the sheet resistance of graphene-based printed e-textiles by three orders of magnitude compared with untreated textiles.	Inkjet deposition, chemically reduction
Salvo [70]	3D printing dry electrodes for ECG/EEG recording	Acrylic-based resin, Au	Surface EEG electrode	Each needle is 3 mm high with base diameter of 600 μm and a tip diameter of about 100 μm, distance of 250 μm, and the impedance at 10 Hz is 62 kΩ	Sputtering, 3D printing
Kaitainen [71]	Liquid silicone rubber (LSR)-based dry bioelectrodes	Conductive liquid silicone rubber, Ti/Ag	Surface EEG electrode	Its impedance might be under 30 kΩ (uncoated) and under 10 kΩ (Ag-coated) at 1–1000 Hz.	magnetron sputtering
Krachunov [72]	3D Printed Dry EEG Electrodes	Printed plastic, Ag/AgCl ink	Surface EEG electrode	Using low cost desktop 3D printers and off-the-shelf components for the fabrication, which allows quick and inexpensive electrode manufacturing and opens the possibility of creating electrodes customized for each user.	3D printing
Lin [73]	Novel dry polymer foam electrodes	Conductive urethane material, conductive fabric, Ni/Cu	Surface EEG electrode	This foam electrode is fabricated by an electrically conductive polymer foam covered by a conductive fabric, the impedance at 10 Hz is 15 kΩ on the hairy site, 8 kΩ on hairless sites.	Not mention
Sinha [78]	Screen-Printed PEDOT:PSS Electrodes	PEDOT:PSS, textile	Surface ECG electrode	After five layers of PEDOT:PSS over an area give a sheet resistance of 5.6 Ω/sq. The SNR of the ECG signal is found to be 15.42 dB under dry skin conditions.	Screen-Printing
Castrillón [79]	PEDOT:PSS-based textile electrodes	PEDOT:PSS, textile materials	Surface ECG electrode	The textile electrodes are fabricated by treating different textile materials with PEDOT:PSS, there is no significant differences in acquiring ECG signals for different materials.	Dipping
De Camp [81]	Light-cured polymer electrodes	PEDOT, polymer	Surface EEG electrode	The electrode get cured by the application of blue light for a few seconds. The impedance was in a range from 10 Hz to 1000 Hz and results in values between 1.2~0.8 kΩ.	Light curing procedure
Bihar [82]	Inkjet-Printed PEDOT:PSS electrodes on paper	Paper, PEDOT: PSS	Surface ECG electrode	The electrode is fabricated by printing PEDOT:PSS on the commercial paper, which is eco-friendly and recyclable.	Inkjet printing
La [83]	Two-Layered and Stretchable e-Textile	Ag particles, fluoropolymer, PDMS, PU	Surface EMG electrode	A two-layered textile electrode is designed by controlled permeation of Ag particles and fluoropolymer composite ink into a porous textile. It has a good conductivity of about 3200 S/cm.	Printing, penetration
Jiang [84]	Polypyrrole-coated nonwoven fabric electrode	PET, nylon, ppy	Surface EMG electrode	This electrode using ppy-coated fabric sheet as conductive layer to realize sEMG acquisition. It can be sewn on the elastic band to guarantee close contact with the skin.	Dipping and coating

**Table A3:** Status of capacitive electrodes.

References	Description	Materials	Type	Abstract	Technology
Chen [98]	Novel Noncontact Dry Electrode With Adaptive Mechanical Design	Copper, plastic, steel spring	Capacitive EEG electrode	The basic scheme contains a metal plate electrode and an active circuit. The compression of the steel spring efficiently reduce the motion artifact.	Not mentioned
Baek [99]	Conductive Polymer Foam Capacitive Electrode	Polyolefine, polyurethane, Au, Ni/Cu	Capacitive EEG electrode	The size for the electrode of 36 mm in diameter by 17.71 mm in height. The impedances of the capacitive electrode is much higher than the Ag/AgCl electrode at a low frequency range.	Not mentioned
